# The Diseases of the Stomach; with an Introduction on Its Anatomy and Physiology

**Published:** 1859-11

**Authors:** 


ART. II.—*The Diseases of the Stomach. With an Introduction on its Anatomy and Physiology; being Lectures delivered at St. Thomas's Hospital.* By WILLIAM BRINTON, M.D., Fellow of the Royal College of Physicians; Lecturer on Physiology and on Forensic Medicine in St. Thomas's Hospital; Physician to the Royal Free Hospital. 8vo., pp. 406. London: John Churchill, 1859.

SHAKSPEARE justly calls the stomach "the storehouse and shop of the whole body." If one organ can be said to be more important than another, we may fairly regard the stomach as the fundamental portion of that complex apparatus whose great function is to provide for the nutrition of the body. It is through the agency of this organ that nutritive material is prepared and supplied to all parts of the economy. It is not only a reservoir for crude alimentary substances, but a laboratory also, in which these materials undergo that change which is necessary to their subsequent assimilation by the blood and tissues. The stomach is the *fons et origo* of the living blood. If its actions be deranged, the life-stream is injuriously affected at its very source. The functions of the stomach are every day being impaired by various circumstances and in various ways; sometimes temporarily and to a slight extent, sometimes chronically and in a serious manner. Partly on account of their inherent importance, and partly because of their influence upon, and intimate relations with, all other maladies, gastric diseases occupy an important place in the nosological scale. Their importance, however, is not due to their frequency and special pathological relations only, but to their obscurity, to the defective knowledge which we possess concerning them, and to the difficulties which surround their study. It is not easy to ascertain the physical condition of the stomach during life. We cannot see it, we cannot distinctly feel it as we can the liver and the spleen; nor can we inspect its secretion, as we can the urine, in a separate and unmixed state. The aids to diagnosis afforded by auscultation in the diseases of the thoracic viscera, and by chemistry in those of the urinary apparatus, scarcely find any parallel in the maladies of an organ which executes its work without perceptible sound or movement, and only dismisses its product from the body after a complex series of changes and admixtures. Hence we are compelled to infer the nature of gastric disorders, not so much from physical evidence, as from the functional disturbance to which they give rise. But the functions of the stomach, as has been well observed, are more readily deranged than those of any other secreting organ, and by a greater variety of conditions. Irritation of the brain, the passing of a gall-stone, obstruction of the bowels, disease of the kidney or of the uterus, will cause vomiting as frequent and distressing as organic disease of the stomach itself. Any sudden emotion, a febrile condition, however induced, various unhealthy conditions of the blood, will suspend or derange the secretion of its solvent juice. Again, the morbid anatomy of the gastric mucous membrane has made less progress than that of some other portions of the body. In the brain, the liver, or the kidney, in the peritoneal, pleural, and other serous membranes, we can readily detect, by post-mortem examination, the traces of the inflammatory action which may have destroyed the life of a patient; and we may, from the careful study of these traces, obtain much valuable information relative to the morbid process of which they are at once the results and the indices. In the gastric mucous membrane, on the contrary, the evidences of disease are, for the most part, more evanescent, and the diseased products effused upon the surface are so intimately mixed with the normal fluids of the stomach and intestinal tract, and with food, drink, and medicine, that they furnish us with very unsatisfactory information of the pathological condition of the membrane upon which they were discharged. Another difficulty in the study of diseases of the stomach, and one which receives but a brief notice at the hands of our author, but is examined at some length in the excellent treatise of Dr. Budd on the "Organic Diseases and Functional Disorders of the Stomach," is the fact that "the stomach presents various appearances, independent of disease, according to the condition of the person when death occurred, and the time of year, and the mode of death, and the time after death at which the body is examined; and several of these appearances are very like the products of diseases, and have, indeed, been generally confounded with them." The frequency and curability of gastric disorders, and the facility with which their symptoms are often recognized, though undoubtedly favorable to the acquisition of information, have nevertheless, as it would seem, misled rather than facilitated stricter pathological inquiries; and have thus rendered our knowledge of diseases of the stomach diffuse rather than profound, and useful rather than accurate.

To diminish this obscurity, and to add to the existing knowledge of these diseases, is the avowed object of the work before us.

This work consists of a series of six lectures, five of which were delivered before "an audience composed of the more advanced students of St. Thomas's Hospital;" while the remaining one (Lecture VI.) was originally "read as a paper before the Physical Society of the same hospital during the session of 1858." To these lectures is prefixed a summary of the anatomy and physiology of the stomach, derived from the physiological lectures of the author, and illustrated with some engravings drawn on wood by himself.

Passing over the anatomico-physiological introduction, we come to Lecture I., which is entirely occupied with the symptomatology of gastric disease. The prominent symptoms of gastric derangement are pain, eructation, regurgitation, vomiting, hemorrhage, and flatulence. These are considered by our author in the order just mentioned, and in a brief but practical manner.

Pain is one of the most striking of the abnormal phenomena exhibited by the stomach, and oftentimes the first to arrest the attention of the patient. It is well known that the stomach is not endowed with that common sensibility which is so well marked in the skin, and which enables the latter to appreciate with such accuracy the mechanical and thermical properties of bodies. Sensibility of this kind in the stomach would be abnormal. Only two sensations appear to be referable to this organ in a state of health—the feeling of hunger periodically calling for food, and of comfort or satiety following its ingestion. The popular notion that a healthy person ought to be unconscious of having a stomach is strictly correct. "The substances he eats and drinks may range from a warmth of 120° to a cold of 32° F.; and may differ scarcely less widely in their physical properties and arrangement. But when once they have traversed his œsophagus, and reached his stomach, these peculiarities are utterly beyond his recognition." Though the stomach is devoid of all common sensibility, it possesses, nevertheless, a special sensibility of its own, and one which, to a certain extent, is independent of the cerebro-spinal centre.

"Closely related to this centre by the feelings of hunger and satiety—nay, more, dictating to it (so to speak) those exertions which the proper alternation of these two states imperiously demand from the mass of mankind—it has a sphere of action altogether its own. And the study of digestion has shown us how admirably and silently the stomach fulfills its various and complex tasks; and how, incidentally to these, the unfelt particle of food no sooner touches its mucous surface, than it excites the flow of a variety of secretions, both far and near, and provokes movements in the muscular substance of its walls and vessels, as well as in the analogous structures of neighboring parts. To these acts, which respectively constitute the sensation and motion of the healthy stomach, its morbid states afford an instructive parallel. And just as the kind of sensibility specific to a healthy muscle—the feeling of its strength, its equilibrium, its measurable force—seems to be traceable, by gradual modifications, through healthy fatigue to the feverish soreness of over-exertion, and through this to the universal muscular pain and prostration of various grave general ailments, so the indistinct sensation of the healthy stomach affords us the best clue to the acute sensibility of the diseased one; and allows us to trace a scale of similar kind—from satiety to repletion; from repletion to distention and weight in the epigastrium; and from hence to the dull, heavy aching of dyspepsia, the gnawing or burning pain of ulcer, and the sharp agony of cancer of the stomach."

Though pain originating in the stomach is often referred to distant organs, as is exemplified in cases of toothache and headache dependent upon irritation of the stomach, yet in the majority of instances it is seated in the epigastric region, and is felt there by the patient. This pain is very protean in its character. "It is often absent when we might fairly expect it to be present; present when we have no clue to any local cause for it; variable or intermittent without any assignable reason; and lastly, even when there is a definite and circumscribed lesion, gives but a wide and indistinct indication of its place, or perhaps refers it to a point of the abdominal surface much above or below, to the right or to the left of its true situation." The indistinctness of the pain is chiefly due to the fact that the stomach is connected with the cerebral perceptive centre by collateral nervous communications and intervening ganglia or independent centres, such as render the transmission of the stimulus far less certain, and its modification during this transit far more probable than would be the case in a stimulated cerebro-spinal nerve. It is due also in part to the situation of the stomach; to the number of organs with which it is in contact; and to the frequent displacements which their movements and its own permit. The exact situation of the stomach is, to a certain extent, altered, and the area of indistinctness of pain thereby increased, by every movement of the heart, lungs, diaphragm, liver, intestines, etc. Besides, as is well remarked by our author, in pointing to the epigastrium the patient is pointing to a kind of focus, formed by the convergence and attachment of a number of important organs, and hence liable to be occupied by the pain which the lesion of any one of them can produce. Pericarditis, pleurisy, gall-stones, hepatic abscess, diaphragmatic lesions, emphysema of the lungs, and a variety of intestinal causes, such as obstruction of the small intestine occupying the right iliac fossa, are all capable of producing what is, strictly speaking, pain in the epigastrium, and, therefore, so far simulative of gastric disease.

Among the most obvious and important causes of gastric pain are undue distention of the stomach, produced by pyloric obstruction, flatulence, etc.; local injury, eroding branches of nerves and irritating their severed extremities, as in gastric ulceration, cancerous deposits, etc. Dr. Brinton thinks that the immediate cause of gastric pain is undue distention of the vessels, irritating, stretching, or otherwise injuring the nerves distributed upon and within their coats. The great changes of calibre which the arteries and veins of the stomach undergo so often, in the sudden transitions of this organ from rest to activity, and *vice versâ*, are doubtless mediated, he thinks, by nerves which supply their muscular coats, and have so far a motor function. He maintains that the violent gastric pains which sometimes occur quite independently of any perceptible lesion, and even (if we may trust the microscope) of any interstitial exudation, are best explained as provoked by a physical irritation or injury of nerves—perhaps sometimes a mere excess of their natural stimulus—infringing rather upon the nerves of submucous situation, distributed to the small arteries and veins of the stomach, than upon any ultimate nervous ramifications in the secretory structures themselves.

The true value or significance of gastric pain is dispatched by our author in the following brief but judicious language:—

"The pain of gastric disease is grave, in proportion not only to its severity, but also to its concentration and fixedness. In other words, a severe and continuous pain, confined to a single spot of small size, is a more serious indication than one which, at times of equal (or nearly equal) severity, fluctuates in its different attacks, and ranges the epigastrium, of which it habitually occupies a wide area. Pain is graver in or near the median line; not only because (for many reasons which will readily suggest themselves) it is, *cœteris paribus*, more certainly gastric here than elsewhere, but because this situation (at least such is my opinion) indicates a more serious derangement of the innervation of the organ than where the pain has a less exact correspondence with the solar plexus. Lastly, of all situations, a median and dorsal one (in anatomical language rachidian, and ranging from interscapular to lumbar,) which is usually an addition and complication to a previous epigastric pain, is the most serious: so much so, that it will rarely be found associated with any but the graver gastric dyspepsias; and belongs chiefly to deep ulcers, or to cancerous lesions of the stomach, involving all its coats.

"In all cases it is essential to add the information obtainable by pressure to the mere sensations complained of by the patient. It is rarely that severe or continuous pain affects the stomach without being associated with some soreness or tenderness to pressure. But when pain is sudden and temporary, and is attended with much flatulence, it is often relieved by gentle pressure. Hence the occurrence of either modification in any given case is capable of greatly influencing our opinion as to the nature of the pain. For, aided by a strict physical examination of the existing state of the stomach in respect of size and situation, it sometimes definitely refers to this organ a pain which, in its absence, could only be conjectured as gastric. In many cases, too, tenderness does far more. According as it is moderate or excessive, superficial or deep, localized or diffuse—features the due recognition of which often imply great delicacy and skill —it justifies a conclusion, not only as to the nature of the gastric malady, but even as to its seat; distinguishes, for example, general inflammation from ulceration or dyspepsia, or a lesion of the peritoneal from one of the mucous coat."

The act of vomiting, the next of the symptoms indicative of gastric disorder, occurs through the intervention of the cerebro-spinal centre, and, as regards its causes, is divisible into two kinds or varieties. In one the irritation provoking the act originates at the nervous centre as in cases of cerebral injury or disease, or mental emotion; in the other, it arises at the periphery, and is thence transmitted to the centre, to be reflected upon the various organs which are concerned in the expulsive effort. Irritation of the fauces, intestines, or peritoneum; disgusting sights, sounds, or smells; prolonged immersion in cold water; and even wounds of the extremities are productive of this kind of vomiting.

"Among the vomitings produced by gastric derangements, we may distinguish the following varieties: Firstly, the vomiting brought about by sheer destruction of tissue, involving an abnormal irritation of the nerves laid bare at the seat of lesion; a variety exemplified in simple and malignant ulceration, in wounds of the stomach, in corrosive poisoning, and characterized (as might be expected) by a remarkable amenability to the physical or chemical properties of substances brought into contact with the injured nerves, (as in the ingestion of food.) Secondly, the vomiting of obstruction, which is referable not so much to the mere obstruction as to the distention and violent muscular movement which is gradually brought about behind the occluded part, and which therefore varies, not only with the strictness of the occlusion, but with its proximity to the pylorus, its superficial extent, its disposition relatively to the muscular coat, and other circumstances of this kind. This variety of vomiting is often seen in cancer and (a still better example) in cicatrized ulcer of the stomach. Thirdly, a kind of vomiting in which the gastric distention present appears mainly referable to a loss of contractile power by the muscular coat of the stomach, (the structure of the organ remaining unchanged.) and in which we must often doubt whether this failure of contractility is not caused by some nervous lesion itself answerable for the vomiting—whether, in short, the distention of the stomach is simply concurrent, or really causative in the process.

"Such allusions to the nature of vomiting may end by the general statement, that, just as it is the kind of vomiting which dictates its treatment—so that, for example, the cerebral vomiting of sea-sickness is often relieved by food and stimulants, the vomitings of phthisis by whatever will diminish or mitigate cough—so the vomiting produced by gastric derangement is most accessible through the function of the organ: in other words, is most amenable to a treatment regulating the quantity and quality of the food, and the frequency of its ingestion."

Hemorrhage, of itself, is by no means a reliable symptom of disease of the stomach. Blood in larger or smaller quantities may be discharged by vomiting from the stomach, or may escape from the intestinal canal, *per anum*, and appear in the fæces without being at all indicative of gastric lesion. The blood which is thus ejected from the economy may have found its way into the alimentary canal as the result of epistaxis, hæmoptysis, gall-stones, hepatic cysts, lesions of the pancreas, etc. It may have escaped from the vessels of the digestive tube in consequence of some mechanical obstruction to the portal circulation produced by cirrhosis of the liver, by tumors or deposits compressing the portal vein, etc. Great care, therefore, is often required in tracing the so-called hæmatemesis or hæmatokopræsis to its proper source and cause. On the other hand, a slight and intermittent hemorrhage into the stomach so often depends on casual circumstances for its detection, that we can never presume its non-occurrence because its results have not been observed. Nothing indeed but a constant and sedulous observation of the gastric and intestinal evacuations affords any valid conclusions of this kind.

In noticing the causes of gastric hemorrhage, Dr. Brinton repudiates the old doctrine of hemorrhage "by exhalation" through the coats of the vessels independently of any solution of their continuity. It is very true, as he remarks, that the finest capillaries have no pores of appreciable magnitude such as would be necessary for the transit of blood-corpuscles. But a passive hemorrhage may be, and in fact is sometimes, due to an exceedingly diffluent state of the blood—the corpuscles escaping not as entire structures, but in a fragmentary or broken-down condition, and mingled with the hæmatin or coloring matter. Such a phenomenon we witness in some varieties of purpura hemorrhagica and scurvy, and in the ecchymoses of malignant fevers. The blood in these cases exhibits under the microscope but few or no red cells.

Scanty extravasations of blood take place in the stomach with great facility, and are attended with but little risk to life. Large effusions of blood, on the contrary, though of rare occurrence, are very dangerous. In explaining these peculiarities, our author points out with care the intimate connection which exists between the anatomy of the vessels of the stomach and the phenomena of gastric hemorrhage.

"The capillaries of the stomach, it will be remembered, are of two kinds: deep, inter-tubular, or gastric proper, as we may call them; and superficial or supra-tubular, which adjoin the general cavity of the organ. In rare instances, the former seem to constitute the chief or exclusive source of hemorrhage, so as to fill more or less of the blind end of a stomach-tube with blood. But hemorrhage is far more commonly confined to the latter—to those complex rings of capillaries which surround the open mouths of the stomach-tubes, and directly underlie the ridges and papillæ intervening between their orifices. These vessels, extremely numerous, and possessing a size and arrangement such as renders them almost intermediate between true capillaries and veins, seem to be peculiarly exposed to the ordinary causes of extravasation. They evidently have all that liability to congestion which belongs to the commencement of the venous part of the circulation throughout the body. They have a situation which requires them to bear the brunt of the various agents introduced into the human stomach in the food. And their delicate membranous wall is only shielded from the physical and chemical action of these agents by a layer of columnar epithelium; the removal of which, though doubtless strictly abnormal, must at any rate be a frequent accident in the history of the digestive process. Indeed, if we think of the incidents of a single meal—the fluids and solids; the animal and vegetable substances; the peppers, acids, wines, liqueurs, spirits; the frozen and scalding, the hard and soft matters which are sometimes brought into rapid and successive contact with the gastric mucous membrane—(and this, too, at a time when the process of digestion itself implies a state somewhat akin to congestion, in so far as it distends and thins these delicate vascular and epithelial structures)—we can easily understand that (as Dr. Beaumont's observations indicate) the soft columnar cells may be here and there stripped off, and the subjacent vessels here and there distended and ruptured, by a casual debauch or dyspepsia, to be restored by a few days of abstinence or temperance to their pristine perfection.

"Little, therefore, can be concluded from the detection of a small quantity of blood in the matters rejected from the stomach of a patient. However suspicious a circumstance, it is only by careful collation with other symptoms that it materially influences diagnosis. It proves a solution of continuity in the vessels yielding it; but leaves us to determine whether this rupture is due to vascular obstruction, to congestion, to desquamation, or to ulceration.

"In larger quantity, gastric hemorrhage has not only more gravity, but a more definite import. Thus, sometimes it presents sufficient of the characters of arterial or venous blood to justify its reference to a vessel of one or other of these two kinds. And even more frequently it shows, by the clots it contains, that it must have been effused rapidly, either from one or two large, or from numerous small vessels. In other cases, it presents the peculiar black color and tarry consistence which blood assumes by exposure to the digestive juices. Since this change of color requires a certain time, as well as a certain amount of these juices, to produce, its occurrence in vomited blood generally indicates that the hemorrhage has either been slowly effused, or is of but moderate amount. In blood discharged from the stomach by the bowels, of course no such rule will obtain; indeed, such a hemorrhage will rarely fail to exhibit somewhat of this tarry color and consistence, unless its quantity have been excessive, or its transit through the intestinal canal unusually rapid. The detection of hemorrhage is sometimes rendered difficult by the admixture of blood with other substances of similar appearance: especially with various articles of food, and with more or less altered bile, occasionally even with morbid products. A careful examination will, however, generally clear up any obscurity of this kind. Indeed, a mere dilution of the inspected matters with water usually suffices; or, if not, a microscopic examination rarely fails to decide the question."

Our author next treats of flatulence, another and a very distressing symptom of gastric disease. The gases found in the stomach and intestines are referable to a variety of sources. Air is habitually introduced into the alimentary canal from without the body, by some persons, voluntarily, as an act of deglutition or eructation; by all persons, in the ordinary act of swallowing, probably in a state of mechanical adhesion to the bolus of food, and certainly in a state of minute division in the frothy saliva. The air thus introduced into the stomach undergoes a process of diffusion or interchange with the elastic fluids dissolved in the blood of the gastric capillaries; a diffusion which converts the ordinary gaseous mixture of the atmosphere into one containing less oxygen and more carbonic acid, and in a degree varying according to the duration of its sojourn in the stomach. The gases found in the intestines are chiefly due, however, to the decomposition of the food which they contain. Occasionally, in the course of certain constitutional diseases, gases are evolved in the digestive tube by the decomposition or putrefaction of the fluids which are naturally poured into that tube. This, however, is a rare source of flatulence in gastric diseases proper. According to a number of writers, another source of flatulence is to be found in a process of gaseous secretion which is effected by the vessels of the alimentary tract. Dr. Brinton denies the occurrence of this secretory process. He reminds us that the gases found in the intestines are, both in quantity and quality, precisely those which would be evolved by the decomposition of the various articles of food, and, under circumstances of complete and sudden starvation, are often entirely absent from a great part of the alimentary canal. He reminds us, furthermore, that some of these gases, such as hydrogen, and carburetted and sulphuretted hydrogen, have never been detected in the blood in any such appreciable quantity as would be necessary to explain their evolution from it. Nor can he find any parallel to such a gaseous excretion even in the case of those structures which, like the lungs, are specially organized with reference to the giving out from the blood of certain of its gases and the taking up of others from the surrounding air. For the gases just alluded to, as absent from the blood, are equally deficient in the air of expiration; nay, more, involve, directly or indirectly, a deoxidation of water, such as is not only without parallel in the chemistry of the organism, but is curiously opposed by that oxidation of hydrogen which forms about a pint of water daily in the healthy human subject; while the carbonic acid and nitrogen common to flatus and expiratory air are scarcely less distinguished by their quantitative relations, than are these others by their presence and absence respectively. In the course of his argument, our author refers to the well-known experiment of Magendie, in which the ligation of an empty intestine in a healthy dog is soon followed by its distention with flatus. The inconclusiveness of this experiment is sufficiently indicated, he thinks, by the fact that it does *not* exclude all the alimentary matter, but that, on the contrary, since one grain of starch or sugar would yield, by decomposition, gases capable of occupying about eight cubic inches of space, no such experiment can exclude the presence of sufficient food to account for the gases of the resulting distention: can approach, indeed, the trustworthiness of the contrary observation, as to the empty and contracted state which often results from starvation.

Lecture II. treats of gastritis, and the various appearances exhibited by the stomach after death. Engorgement and hypostasis of the stomach, alterations in the color of its lining membrane, produced by the infiltration or imbibition of biliary pigment and the various coloring matters derivable from food and medicine, such as the red color of wine or logwood, or the black color of the metallic sulphurets reduced from the solutions of their salts; changes in the shape and size of the stomach and the thickness of its coats; mammillation, effusion, post-mortem erosion, and solution of the gastric coats, are severally considered by our author in a concise and lucid manner.

The state called mammillation of the stomach is thought by most writers to be produced by an accumulation of glandular or fluid contents within the gastric tubes. Dr. Brinton has convinced himself that this view of its nature is untenable.

"For it not only occurs in persons dying suddenly during health, but also in stomachs otherwise perfectly healthy. It is rarely accompanied by any change in the bulk or appearance of the secretory structures. It affects chiefly the more muscular pyloric half or two-thirds of the organ. Its projections often merge, by slight gradations of size, into wrinkles closely akin to those formed in the healthy and contracted organ. It may always be effaced by pressure, generally by prolonged dilatation. And lastly, though, when of limited extent, it is compatible with a relaxation of some other part of the stomach, it is generally associated with local contraction, and is never found both marked and extensive, save in a contracted stomach. As respects the mode in which it is brought about, I believe it to be due, not to a mere contraction of the longitudinal and transverse layers of the general muscular coat—as is evidently the case with the ordinary *rugæ*—but to a much more irregular and uncertain contraction specially shared in by the various bundles given off from this coat to form the muscular constituent of the matrix, in which the tubes of the mucous membrane are packed vertically side by side. In short, it seems to be somewhat analogous to those curious distortions by which the villi share in the *rigor mortis* of the muscular coats of the intestine. I may add, that the permanence of the phenomenon seems to be sometimes furthered by a subsequent process of transudation, which distends the submucous areolar tissue of the various isolated projections or *mammillæ*, with a serous fluid of the same kind as that often found occupying a larger extent of the cellular coat."

The larger portion of this lecture is devoted to a very judicious account of the symptoms, pathology, and treatment of gastritis; and catarrh, hemorrhagic erosion, and follicular ulceration of the stomach.

Lecture III., which occupies about one-fourth of the whole volume, is derived chiefly from an excellent monograph on Ulcer of the Stomach, published by our author in 1857. As this monograph was reviewed in Vol. II. No. 6, of this Journal, we refer our readers to that number for an account of the views of our author relative to this interesting disease.

Lectures IV. and V. are occupied with the consideration of cancer of the stomach; cirrhotic inflammation or plastic linitis, suppurative linitis, tumors, hypertrophy and atrophy of this organ; dilatation of the stomach, resulting from obstruction, destruction, injury, and paralysis; and secondary inflammation. Our limited space will not permit us, however much we might desire, to place before our readers a résumé even of the valuable facts and arguments which Dr. Brinton has been at much pains to bring together, with a view to elucidate the nature and pathology of the disorders above mentioned, and to found thereupon a strictly philosophical and successful treatment. Suffice it to say, that these lectures will repay a careful perusal; and we confidently refer the reader to them for much valuable information, both practical and eminently suggestive in character.

Dyspepsia constitutes the subject-matter of Lecture VI. and last. Dr. Brinton has evidently studied the protean phenomena of this distressing functional disease with the greatest care. He examines first what he calls the gastric and intestinal relations of the disease. Disturbance of the gastric functions is by no means the exclusive cause of those symptoms which, in the aggregate, constitute what is called dyspepsia. There are many cases of this disease in which the stomach is by no means the organ first and most in fault. Indeed, it would appear, from the observations of Dr. Brinton, that in many cases of dyspepsia, the stomach is perfectly capable of executing any reasonable amount of work.

"But such a conclusion by no means disproves the close relation of dyspepsia to derangement of the stomach, or excludes it from consideration among the diseases of this organ. It may be quite true that, in some cases, the stomach is only distressed by the excessive amount of food introduced into it; and is only injured by being overtasked, much as a muscle or a tendon might be. It may be equally true that, in others, it is weakened by want of exercise, or deranged by a surplus of the materials amenable to other parts of the digestive process; that it languishes for want of the protein compounds it ought to elaborate; or is surcharged by starchy or fatty matters foreign to its office—perhaps often in excess of what can be assimilated by the organs whose function it is to do so. Still since, in a vast majority of cases, the symptoms of dyspepsia are referable mainly to the stomach—since the organ is either primarily or secondarily deranged, and the symptoms of that derangement are the chief phenomena observed by the physician and felt by the patient—we may safely accept the ordinary view, that dyspepsia generally represents a functional malady of the stomach. Doubtless there are many derangements of other parts of the alimentary canal equally entitled to the name of indigestion. But with the exception of intestinal dyspepsias attended by diarrhœa—and often accompanied by such a catarrhal state of the large intestine as amounts to a structural lesion—the symptoms strictly intestinal are generally scanty and obscure, in comparison with those traceable to the stomach. And hence these latter may justifiably be the chief objects of our study. Even were they mere incidents of the malady, so long as we remembered that they were not its essential or sole features, but only those most accessible to our scrutiny, we should be entitled in according them a notice proportionate to their importance. But when we find (as we do) that the stomach not only always shares, and sometimes almost monopolizes, the derangements called dyspepsia, but that it is the chief source of their most striking symptoms, and that it is also more amenable to treatment than any other part of the digestive canal,—we are justified in describing dyspepsia as being, for practical purposes, a gastric disease.

"Why the symptoms of dyspepsia refer chiefly to the stomach it is not difficult to understand. The physiology of digestion affords us plausible grounds for presuming that the details which distinguish this organ from the remainder of the alimentary canal—especially its shape, its size, its situation, its office, and the peculiarities of innervation associated with them—give it a kind of paramount importance, and render it, in the main, far more sensible to various disturbing agencies, and far more disposed to betray disturbance by abnormal phenomena—such as pain, vomiting, or flatulence—than any other segment of the digestive tube. So that even were purely intestinal dyspepsia much more frequent and important than it seems to be, the study of gastric dyspepsia would still be the best means of approaching its consideration. Apart from the frequent secondary involvement of the stomach in the intestinal variety, the symptomatology of dyspepsia of both kinds would be best studied from its most distinct and accessible side. In this respect, indeed, the functional derangements of the stomach and intestine do but parallel their structural diseases: in which we often find that lesions, otherwise precisely identical, are betrayed by much more distinct symptoms when located in the stomach than in the bowels; and that, in the latter situation, they are sometimes mistakenly referred to the stomach, owing to those secondary derangements of this organ which they are liable to excite.

"Such a view of dyspepsia suggests two considerations as to its diagnosis. Since it represents not so much a single and substantive disease as a variety of ailments, and is characterized not so much by the presence of certain symptoms as by the absence of structural lesions, the diagnosis of the malady in general, and of any case of it in particular, are opposed by analogous difficulties. As the progress of scientific medicine has gradually revealed the morbid anatomy of the digestive canal, and thus detected structural disease with increasing accuracy and frequency, the vague (but useful) term 'dyspepsia' has acquired a continually more restricted meaning. Nor can we doubt that it is destined to still further limitation; and that, as advancing knowledge brings us better means of investigation, and so enables us to discover and distinguish structural changes of which we now can only observe the functional results, the aggregate of maladies called dyspepsia must undergo successive subtractions, tending more or less completely to its total subdivision into special maladies, and to the removal of this term from our nosology. And while I hope that many of you will hereafter repeat (as you advance in the knowledge of your art) this increasingly restricted application of the term 'dyspepsia,' I venture also to predict that, even in the investigation of a single case, you will often find yourselves deducing its dyspeptic nature rather by a gradual exclusion of what it is not, than by any such a sudden recognition of what it is, as, in the substantive diseases of many other organs, often anticipates the result of a systematic inquiry. And (as a kind of corollary to this) let me suggest that not only does 'dyspepsia' still probably include many really structural (if not chronic or permanent) maladies of the organs it affects, but that so closely do its absolute symptoms resemble those of graver diseases, that its importance is enhanced by the possibility of mistaking, for it, such lesions as cancer or ulcer of the stomach. Sometimes, indeed, such errors are almost unavoidable. But, more frequently, they arise from a forgetfulness of the slight characteristics of these structural diseases in their earliest stages; leading to a hasty conclusion, which a brief suspension of judgment would have prevented, or even reversed. Nor is the stomach the only organ whose lesions may be mistaken or regarded as mere dyspepsia. Just as there is none whose diseases do not react upon the organism generally, and thus influence the nature and amount of food the stomach requires, and the instinctive sensations of hunger or anorexia associated with the fluctuations of these wants—so it is hardly too much to assert that every grave constitutional malady has its own dyspepsia, brought about by conditions strictly analogous to those which produce it as an independent ailment, and capable (in those cases in which it is unusually early or prominent) of masking any slight indications of organic mischief in the particular organ to whose lesion it is indirectly due. Much as the progress of physical diagnosis has done for modern medicine, the warnings of our predecessors as to the danger of mistaking phthisis and other diseases for the dyspepsia secondary to them, are even now worthy of your recollection--not only because they afford the strongest motive for a thorough inquiry into the condition of all suspected organs in every case of dyspepsia, but also because the physical signs of these diseases are, in rare instances, so indistinct, as to leave us almost exclusively to that keen scrutiny of symptoms, and that mental process of induction, by which the sagacity of the older physicians sometimes arrived at a diagnosis scarcely less accurate than what is now more easily obtained from physical investigations."

After describing the rise, progress, and symptoms of an ordinary dyspepsia, Dr. Brinton passes in review some of the varieties of this hydra-headed disorder—such as the "flatulent dyspepsia," characterized by severe or continuous pain in the epigastric region; "intestinal dyspepsia," whose specific feature is constipation, alternating with attacks of diarrhœa; "ingestive dyspepsia," in which indigestion closely follows the introduction of food into the stomach; "post-digestive dyspepsia," occurring simultaneously with the propulsion of the contents of the stomach into the duodenum; and "fasting dyspepsia," existing during the intervals of gastric activity, and ceasing upon the ingestion of a new meal. With regard to the classification of the various forms of dyspepsia, our author proposes several valuable suggestions, based upon physiological grounds. Keeping in view the well-known classification of aliments laid down by Prout, he recommends "a division of dyspepsias according to that alimentary constituent, the failure of which to be digested provokes their symptoms." He speaks of the "proteinous" dyspepsia common among the affluent and sedentary, and characterized by headache and epigastric pain, rarely severe, but often referred to the region behind the sternum, corresponding to the cardiac aperture of the stomach; the "starchy" dyspepsia of the poorer classes, chiefly distinguished by the extreme flatulence which generally accompanies it; the "oily" dyspepsia, attended, in addition to much headache and epigastric pain, with severe and continuous nausea or even vomiting. Dr. Brinton thinks that even "saline" and "aqueous" dyspepsias might be added to this list. He has known instances in which persons appeared to be rendered singularly dyspeptic by the saline contents of a hard or calcareous drinking-water; as well as others in which nothing but a strict limitation in the quantity of this diluent, however pure, habitually consumed with their food, sufficed to ward off the malady. Of course similar effects may often be traced to the condiments, the peppers and spices, for example, taken with food. But many of these stimulant and acrid substances really possess noxious characters; and few of them can be fairly regarded as ranking among the physiological constituents of food; besides, their ill effects are but secondary and collateral, being referable to the undue quantity of food which they tempt those who use them to consume.

Another classification or grouping of gastric dyspepsia is suggested by our author, based partly upon the chemical reactions of the fluids expelled from this organ by regurgitation or vomiting, and partly upon the physical properties and microscopic appearances of such fluids.

The causes and treatment of dyspepsia are discussed at some length. Counter-irritants are regarded by Dr. Brinton as useful chiefly in the severer forms of the disease, attended with great pain. Pepsine has disappointed him in most of the cases in which he has tried it. Occasionally, indeed, he has seen it produce considerable disturbance of the stomach, even in cases which were carefully selected, and in which there appeared to be no great irritability of the organ. To obviate some of its disadvantages he proposes to use it in the shape of peptone, a form in which he believes it would be of great benefit as a highly nutritive drink or enema in certain œsophageal and gastric lesions. Tonics are regarded by our author as an almost indispensable part of the treatment, unless contra-indicated by gastric irritability. Of all the bitter tonics, quinine is perhaps more frequently useful than any other. As a rule, Dr. Brinton thinks that these tonics should be administered, either on an empty stomach, or, if the appetizing effect is desired, shortly before a meal; but when combined with alkalies, they may often be taken with advantage toward the close of the digestive act. In cases where dyspepsia is complicated with diarrhœa or pyrosis, the more astringent vegetable tonics, such as hæmatoxylon, kino, catechu, krameria, etc., may be used with advantage. Of the various metallic substances employed with benefit in dyspepsia, iron and zinc are perhaps the most useful. Of all the preparations of iron, the citrate is borne by the stomach better, perhaps, than any, especially if administered in an effervescing form. The carbonate, phosphate, sulphate, and sesquichloride are preparations which agree with most stomachs. The efficacy of iron appears often to be heightened by combination with zinc. The oxide and sulphate of the latter substance sometimes prove very useful in the treatment of the disease under consideration. "Bismuth, some of the effects of which may perhaps be regarded as tonic, is still more useful as a remedy against that form of dyspepsia which constitutes the 'morbid sensibility of the stomach' specified by the older writers. Here its effects in allaying flatulence and nausea, and in preventing vomiting, and (still more) in checking the pain produced by food, are so marked, that we may fairly accept the term of sedative often applied to it." The alkalies and their carbonates, and the alkaline earths, are of use chiefly in those cases where flatulence, regurgitation, and heartburn occur at the close of the digestive act. They serve to neutralize the lactic and acetic acids generated by the decomposition of undigested food. They also appear to remove the uric acid diathesis which sometimes complicates the more obstinate varieties of dyspepsia, and influence, perhaps, the hepatic, pancreatic, and intestinal secretions. Acids, besides acting as tonics, seem to increase not only the quantity, but also the solvent properties of the gastric juice. They should be given during or immediately after a meal. The hyposulphite of soda is favorably noticed by our author as a remedy in cases of flatulent dyspepsia. It acts locally upon the stomach, and seems to check decomposition in the food. In some respects it resembles the alkaline carbonates, and may be administered in the same doses and combinations, and with the same limitations. Dr. Brinton recommends the iodide of potassium, in doses of two grains, combined with from seven to ten of the bicarbonate of potassa, in those cases of flatulent dyspepsia in which—whether from a too starchy diet, deficient or hasty mastication, decayed teeth, the abuse of tobacco, or other causes—the salivary secretion seems either deficient in quantity or faulty in quality.

But our limits, already exhausted, will not permit us to follow our author in his remarks upon the use of sedatives, purgatives, mercury, strychnia, silver, arsenic, prussic acid, alcohol, and other articles, in dyspepsia; nor can we reproduce his suggestions respecting the dietetic and hygienic management of this disease. For these remarks and suggestions we must refer our readers to the work itself, a work in which they will find much that is valuable and interesting, and of which we have endeavored to present an analysis as clear and instructive as is consistent with the brevity enjoined upon us.

